# The Recent Veto of the Osteopathy Bill

**Published:** 1900-02

**Authors:** 


### *EDITORIAL NOTES AND COMMENTS.*

---

The Business office of THE JOURNAL-RECORD is 305, 306 Fitten Building.

The Editorial office is 318-319-320 The Prudential.

Address all Business communications to Dr. M. B. Hutchins, Mgr.

Make remittances payable to THE ATLANTA JOURNAL-RECORD OF MEDICINE.

On matters pertaining to the Editorial and Original Communications address Dr. Bernard Wolff, Atlanta.

Reprints of original articles will be furnished at cost price. Requests for the same should always be made *on the manuscript.*

We will present, post-paid, on request, to each contributor of an original article, twenty (20) marked copies of THE JOURNAL-RECORD containing such article.

---

### THE RECENT VETO OF THE OSTEOPATHY BILL.

WE understand that Governor Candler has received many letters of adverse criticism upon his recent action in regard to the *osteopathy bill.* We have also seen some similar criticisms in the public print purporting to have been made by a *a so-called ministerial divine* from an Atlanta pulpit. The latter also preached a sermon (?) to the students of medicine attending school here in the city, during the course of which he gave some advice to these young men which, if followed out, would make them joint heirs with his satanic majesty, and heap upon them while living all the opprobrium which self-respecting people could give. It is certainly a blot on the medical profession that this same divine can sign his name with an M.D., and claim the high honor of holding a membership in the North Carolina Medical Association. However, these side remarks are secondary to the real intent of this article. The time has come when physicians must stand together as one solid phalanx, and by this unity of purpose aid in the control of legislation which must either be for the elevation or degradation of the medical profession. Politics is the ruination of our country, and needed legislation is throttled in its very incipiency by those who hope to make political gain by such action on their part. The physicians of this State are decidedly an integral part of the voting population, and adding to this their personal influence, the time has come when they should make this power felt in electing the proper men to our State legislature. We do not favor a political conclave, but we do favor a united and concerted action on the part of physicians throughout the State in aiding the election of men of intelligence and high moral character. As was recently said in an editorial of the Cincinnati *Lancet-Clinic*, the "barriers are fast being burned away" and the differences between the three recognized schools of medicine are fast becoming a thing of the past. They all have the same aims, use the medicines from the same *materia medica*, and, above all, each is trying to elevate the cause of medical education in our various schools. When a delegation of physicians appeared before the Governor to urge him to exercise his veto power upon the "Osteopathy Bill" the three schools of medicine were all represented, and each recognized that legislation which affected one must affect the other. Certainly the field is broad enough as represented by the three schools to allow any man an opportunity to practice medicine within the State. Governor Candler's stand in vetoing the bill passed by the legislature for a private enterprise, and his intelligent message showing that there was already sufficient medical legislation in the State, deserves hearty commendation from the profession throughout Georgia. Not only does his action deserve it, but the medical profession will see that he gets it, for we must all recognize the unusually clear intelligence of our chief executive. In future years if Governor Candler should ever need the support of the medical profession in this State, let us pledge that he shall have it, and return to him some of the great benefit that he has done to the public by trying to elevate and not to debase one of our noblest professions. While we are commending this action of the Governor we cannot but condemn that of others who claim to be our medical brethren. We feel that the physicians throughout this great State should know the names of those doctors of medicine who, as members of the last legislature, not only did not raise a voice in opposition to the passage of the osteopathy bill, but actually came to its support. Think of medical men trying to throttle the very profession which has given them their start in life! While the medical men in Georgia may not in themselves be able to change materially the tone of the legislature, they, however, can have a great influence in defeating those men who are trying to degrade medicine in Georgia.

A little history of the late osteopathy bill may not be amiss, since the battle has only begun, and physicians throughout the State must recognize the necessity of union if they hope for ultimate success. The bill was introduced by Senator Nesbitt, from the Atlanta district; thence it was referred to the General Judiciary Committee. This committee reported it back favorably to that body. At the request of the chairman, Dr. Baird, of the Committee on Medical Legislation from the Georgia Medical Association, the bill was recommitted to the General Judiciary Committee in order to hear arguments from the medical men in opposition to its passage. At the conclusion of these arguments, it was stated that they had made the case so clear that it would be reported back unfavorably, and it was so announced in the afternoon daily. Yet, in spite of this, the committee two days afterwards unanimously reported the bill back to the Senate, and it was then passed. From the Senate the *osteopathy bill* went to the lower house, and was there referred to the General Judiciary Committee of that body. This committee, wishing to be relieved of the opprobrium of fostering and advocating the passage of this bill, had it referred to the Committee on Hygiene and Sanitation. Dr. Baird also requested of this committee that the physicians be heard in opposition to the bill before making a final report. This was promised, and yet the bill was returned to the house with the unanimous recommendation for its passage without ever hearing from the physicians in opposition. This committee afterwards said that they reported favorably on the bill *in deference to the Senate.* Such is the intelligence and wonderful magnanimity of our legislature! We think it would be well for the members of the Georgia Medical Association to know that there were five physicians on this committee which reported favorably on the bill, and if they raised a voice in opposition to the passage of this measure we have yet to hear of it.

The Committee on Hygience and Sanitation was as follows:

Dr. G. W. Drawdy of Jesup,
Dr. Chas. W. Howard, Jr., of Cusseta,
Dr. C. H. Turner of Conyers,
Hon. J. F. Harris of Pavo,
Dr. J. W. Mayson of Decatur,
Dr. R. Y. Rudicil of Trion,
Hon. W. H. Cook of Cooksville,
Hon. J. T. Johnson of Smithville,
Hon. J. C. Jarnagin of Warrenton,
Hon. M. L. Hathcock of Ralph,
Hon. E. B. Gresham of Walter,
Hon. A. J. Brown of Maulden Branch,
Hon. A. B. Dickey of Mineral Bluff,
Hon. A. C. Taylor of Smithville. R.

